# No reduction in motor‐evoked potential amplitude during the rubber hand illusion

**DOI:** 10.1002/brb3.3211

**Published:** 2023-08-07

**Authors:** Arran T. Reader, Sara Coppi, Victoria S. Trifonova, H. Henrik Ehrsson

**Affiliations:** ^1^ Department of Psychology Faculty of Natural Sciences University of Stirling Stirling UK; ^2^ Department of Neuroscience Karolinska Institutet Stockholm Sweden

**Keywords:** body ownership, motor control, multisensory integration, transcranial magnetic stimulation

## Abstract

**Introduction:**

In the rubber hand illusion (RHI), touches are applied to a fake hand at the same time as touches are applied to a participant's real hand that is hidden in a congruent position. Synchronous (but not asynchronous) tactile stimulation of the two hands may induce the sensation that the fake hand is the participant's own. As such, the illusion is commonly used to examine the sense of body ownership. Some studies indicate that in addition to the subjective experience of limb ownership reported by participants, the RHI can also reduce corticospinal excitability (e.g., as reflected in motor‐evoked potential [MEP] amplitude) and alter parietal‐motor cortical connectivity in passive participants. These findings have been taken to support a link between motor cortical processing and the subjective experience of body ownership.

**Methods:**

In this study, we tried to replicate the reduction in MEP amplitude associated with the RHI and uncover the components of the illusion that might explain these changes. As such, we used single‐pulse transcranial magnetic stimulation to probe the excitability of the corticospinal motor system as participants experienced the RHI.

**Results:**

Despite participants reporting the presence of the illusion and showing shifts in perceived real hand position towards the fake limb supporting its elicitation, we did not observe any associated reduction in MEP amplitude.

**Conclusion:**

We conclude that a reduction in MEP amplitude is not a reliable outcome of the RHI and argue that if such effects do occur, they are unlikely to be large or functionally relevant.

## Graphical abstract

The rubber hand illusion was induced through visuotactile stimulation of the real hand and a congruently placed rubber hand. Electromyography was recorded from the first dorsal interosseous during single‐pulse transcranial magnetic stimulation. Synchronous visuotactile stimulation (rightSync), which induced the illusion, did not result in a reduction in motor‐evoked potential amplitude, compared to the control condition using asynchronous visuotactile stimulation (rightAsync).

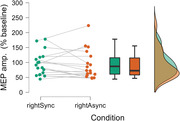

## 1 INTRODUCTION

When we perform a movement, we have a clear sensation that the body we see before us is our own. This sense of body ownership is believed to stem from multisensory integration (Blanke et al., 2015; Ehrsson, 2020; Kilteni et al., 2015): We can see and feel our hand moving, as well as perceive tactile sensation when we interact with objects, and these sensory impressions are automatically combined into a unitary experience of the limb. Thus, by combining these sources of sensory information, the brain's perceptual system can generate an experience of the hand as being one's own. The rubber hand illusion (RHI) emphasizes this by showing how manipulating multisensory information may lead to a sense of ownership over a false limb. When a false hand and a participant's real, hidden hand are stroked synchronously (but not asynchronously), it is possible to induce the sensation that the false hand is part of the body. Aside from this sense of ownership over the fake hand, the RHI is associated with a spatial shift of tactile sensations from the real to the rubber hand (“referral of touch”) and changes in the perceived position of the real hand (proprioceptive drift; Botvinick & Cohen, 1998; Tsakiris & Haggard, 2005). The RHI may also induce feelings of disownership for the hidden real hand (Longo et al., 2008, 2009; Preston, 2013; Reader et al., 2021) when it fades from awareness as the rubber hand is experienced as one's own, though these experiences are not usually so vivid as referral of touch and hand ownership in most participants.

While movements may contribute to changes in the sense of body ownership (Bassolino et al., 2018; Burin et al., 2015, 2017; Fiorio et al., 2011; Kalckert & Ehrsson, 2012, 2014; Longo & Haggard, 2009; Pyasik et al., 2019; Scandola et al., 2017; Tidoni et al., 2014; Tsakiris et al., 2006; but see Teaford et al., 2023), how they do so is a matter of debate, and a clear role for the motor system in body ownership is yet to be established. One view holds that somatosensory feedback from movement contributes to body ownership (only) through multisensory integration with visual and other types of sensory feedback (Kalckert & Ehrsson, 2012, 2014). Others claim that the feeling of being in control of the movement (sense of agency) influences body ownership (Tsakiris et al., 2006), for example, through efferent information from motor commands influencing visuoproprioceptive integration of hand signals (Abdulkarim et al., 2023). Others still have argued for a functional reciprocal relationship between body ownership and the motor system, whereby reduced capacity for movement, either through paralysis, limb immobilization, or non‐invasive neurostimulation, can alter susceptibility to body ownership illusions (Burin et al., 2015, 2017; Fossataro et al., 2018). According to the latter view, the motor system is directly involved in body ownership and the elicitation of the RHI, even under conditions when participants are passive as in the classical version of the RHI.

Less commonly discussed is the potential role of body ownership in motor control (i.e., the inverse of the aforementioned relationship). Though body ownership illusions like the RHI may interfere with goal‐directed actions (Heed et al., 2011; Kammers et al., 2010; Newport & Preston, 2011; Newport et al., 2010; Rossi Sebastiano et al., 2022; Zopf et al., 2011; but see Kammers, de Vignemont et al., 2009; Kammers, Longo, et al., 2009, 2009), possibly by updating the “internal state estimate” of the body that is used by forward models in motor control (Kilteni & Ehrsson, 2017; Wolpert et al., 1998), their possible influence on basic movement (i.e., those performed without objects in body‐centered space) and motor physiology is not yet clear. Experimentally, manipulating body ownership appears to have no influence on basic movements like finger abduction (Reader & Ehrsson, 2019; Reader et al., 2021). However, work using transcranial magnetic stimulation (TMS) provides some evidence that body ownership illusions influence motor processing in the brain (Dilena et al., 2019; Golaszewski et al., 2021). For example, the RHI might alter parietal‐motor cortical connectivity (Isayama et al., 2019; Karabanov et al., 2017) and short‐ and long‐latency afferent inhibition (Isayama et al., 2019). Changes have been observed in motor cortical excitability, as reflected in short‐interval intracortical inhibition (Alaydin & Cengiz, 2021), and one study reported that an “illusory amputation” induced by virtual reality can reduce the excitability of motor circuits controlling the affected limb (Kilteni et al., 2016).

An influential article by della Gatta et al. (2016) similarly reported that a reduction in corticospinal excitability occurs during the RHI. della Gatta et al. (2016) applied single‐pulse TMS over the primary motor cortex to examine the size of motor‐evoked potentials (MEPs) recorded from the first dorsal interosseous (FDI) muscle in 24 illusion‐susceptible individuals at baseline, during the RHI induced by synchronous stroking, and during a control condition with asynchronous stroking. They found that when MEPs in the right FDI were elicited through stimulation of the left (contralateral) motor cortex, and the illusion was induced on the right hand, peak‐to‐peak MEP amplitude was reduced in the synchronous condition, compared to baseline and the asynchronous condition. Furthermore, this effect appeared to increase with time as the illusion was continually induced. Similar results were not observed in a different group of 20 participants when MEPs were recorded from the left hand, for which the illusion was not induced. della Gatta et al. (2016) suggested that a reduction in corticospinal excitability occurred due to disownership of the real hand during the illusion: “If I believe that the hand is mine, then I must be ready to use it; if not, then the activity of the motor system is accordingly down‐regulated” (p. 8). However, the authors did not assess the subjective experience of disownership in the participants for which they recorded MEPs, meaning that they were unable to provide direct evidence for this assertion. Similarly, statistically significant correlations were not observed between MEPs and proprioceptive drift or statements addressing the sensation of ownership over the rubber hand (though behavioral and physiological measures were collected during separate sessions). This weakens the evidence for a link between specific aspects of the RHI and the reported changes in corticospinal excitability.

Further experimentation would be useful to validate the findings of della Gatta et al. (2016), which could potentially suggest a role for low‐level interactions between the conscious experience of body ownership and motor processing. In addition, if such an effect does occur, it is essential to understand why. This might help us better understand the potential motor consequences of bodily awareness disorders (e.g., Pacella et al., 2019; Vallar & Ronchi, 2009), as well as assist the development of prosthetics (Niedernhuber et al., 2018). It is also important for the field of body representation to examine the robustness of this effect since it has theoretical implications for models of body ownership. A negative result would be similarly interesting because it would be in line with multisensory models of the RHI and body ownership that do not include motor processes or the primary motor cortex as a critical structure (Chancel, Iriye, & Ehrsson, 2022; Ehrsson, 2020; Fang et al., 2019; Guterstam et al., 2019; Kilteni et al., 2015; Samad et al., 2015). Notably, Karabanov et al. (2017) did not observe a reduction in MEP amplitude as a consequence of the RHI, though they had a smaller sample size than della Gatta et al. (2016) and used a slightly different paradigm (a moving version of the RHI).

The purpose of this experiment was twofold. First and foremost, we aimed to test the hypothesis that the RHI results in a reduction in corticospinal excitability as reflected in MEP amplitude and reported by della Gatta et al. (2016). This can help to verify the possible influence of body ownership changes on the motor system. Second, if we replicated the effect, we aimed to build on these findings by assessing whether different components of the RHI correlate with change in MEP amplitude to learn more about what is potentially driving the effect. If the subjective RHI is the factor driving such changes in corticospinal excitability, one may expect correlations with the ratings of one or more of the specific items in the questionnaire that reflect the various phenomenological aspects of the illusion (illusory rubber hand ownership, referral of touch, disownership of the real hand, agency); if the recalibration of vision and proprioception is a critical factor, one could expect a correlation with proprioceptive drift. To examine these possibilities, we performed a single‐pulse TMS experiment to probe corticospinal excitability as a group of healthy participants experience the RHI quantified with a questionnaire and proprioceptive drift.

## 2 METHOD

The procedure, hypotheses, data preprocessing, and analysis were registered prior to data collection (https://doi.org/10.17605/OSF.IO/PM5GR). Any changes to this plan or addition of exploratory post hoc analyses are stated below.

### 2.1 Power analysis and stopping protocol

We performed a power analysis based on the results of della Gatta et al. (2016) in G*Power 3.1 (Faul et al., 2007). The smallest effect size estimate provided in their article was *dz* = 0.74, for a difference in MEP amplitude between baseline and synchronous stroking of the rubber hand. Using this effect size we generated a required sample size for 90% power using a one‐tailed *t*‐test at *α* = .05. This resulted in a suggested sample size of 18. This was our preliminary sample size.

If we did not replicate the effect of della Gatta et al. (2016) using a frequentist statistical approach, we planned to assess the level of evidence in favor of the null hypothesis using an informed Bayesian analysis (details below). We planned to collect data until analysis suggested greater support for the null hypothesis versus the alternative or until we reached a total of 30 participants. If we did replicate the effect of della Gatta et al. (2016) using a frequentist statistical approach, we planned to assess whether any components of the RHI correlated with the observed effect. Since we had no feasible effect size estimate for these correlations, we planned to use an uninformed Bayesian approach (details below), collecting data until a majority of correlations provided evidence in favor of the alternative hypothesis versus the null, or vice versa, or until we reached a total of 30 participants.

### 2.2 Participants

We recruited right‐handed participants aged between 18 and 45 from Karolinska Institutet and the surrounding area. Participants were only tested if they were susceptible to the illusion (similar to della Gatta et al., 2016; see below) and if they met the inclusion criteria for TMS (see below). Ethical approval for the experiment was granted by The Swedish Ethical Review Authority (https://etikprovningsmyndigheten.se/, approval #2019‐01216), and participants provided written informed consent. Participants received a cinema ticket for attending the experiment screening and 625 SEK for taking part in the full experiment. The sample used for statistical analysis consisted of 18 individuals (10 women, eight men), aged between 19 and 37 years. The mean ± SD age was 26.1 ± 5.48 years.

### 2.3 Materials

#### 2.3.1 RHI

**FIGURE 1:**
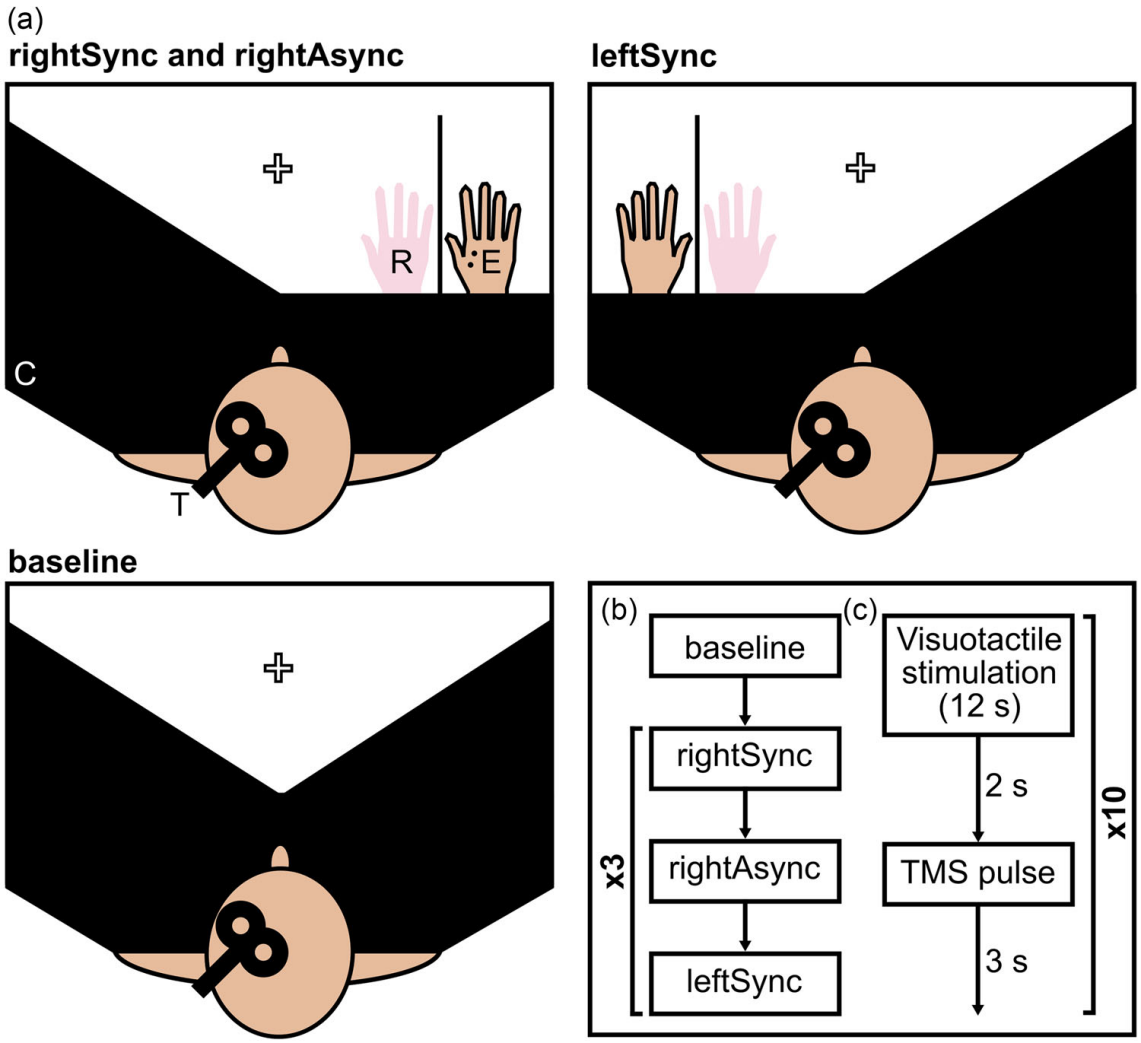
Experimental setup and procedure. (a) Experimental setup for each condition and baseline, C: cloth, T: transcranial magnetic stimulation (TMS) coil, R: rubber hand, E: electromyography (EMG) from first dorsal interosseous (FDI). Note that EMG is always recorded from the right FDI even when the right hand is hidden by the cloth. (b) Example experimental procedure. Condition order was counterbalanced across participants. (c) Visuotactile stimulation and TMS application during each condition run. Proprioceptive drift was recorded before and after this timeline. Questionnaire statements were recorded after this timeline.

Two experimenters performed the experiment: one to induce the RHI and one to apply TMS. Participants sat comfortably at a table. In our preregistration, we proposed that all participants would sit with their heads relaxed in a secure foam‐lined headrest, but five participants did not use the headrest due to changes in the availability of equipment following a delay to data collection arising from the COVID‐19 pandemic. In the center of the table was a white fixation cross. A black cloth and/or an L‐shaped wooden screen was used to obscure the real hands when necessary in the different experimental conditions (see below). The wooden screen was 60 cm long in total, 54 cm high (and 18 cm long) nearest the participant and 31 cm high nearest an experimenter sitting opposite the participant. When a baseline measurement of corticospinal excitability was recorded, both of the participant's real hands were placed on the table, with a cloth obscuring both their hands and their upper body (Figure 1). In experimental conditions, both of the participant's real hands were placed on the table, and either a left (leftSync condition) or a right (rightSync or rightAsync conditions) cosmetic Caucasian male prosthetic hand (Fillauer LLC) filled with plaster (the “rubber hand”) was placed on the table, lateral to the tested real hand and aligned with the participant's shoulder (Figure 1). The screen was placed between the tested real hand and rubber hand, and the participant's other real hand was covered with a cloth, along with their upper body. The middle finger of the tested real hand was placed 10 cm away from the screen. The rubber hand was placed with the middle finger 10 cm away from the other side of the screen (20 cm away from the tested real hand).

In order to assess subjective experience during the illusion, participants were presented orally with statements to which they provided their level of agreement (+3, “*strongly agree*” to −3, “*strongly disagree*”). These questionnaire items were partially adapted from previous work (Botvinick & Cohen, 1998; Longo et al., 2008) and addressed referral of touch, the sense of ownership over the rubber hand, sense of agency over the rubber hand, a feeling of disownership for the real hand, and a control statement for task demands (Table 1). Statement S4 was chosen to assess a sensation of disownership of the real hand in keeping with some previous studies (della Gatta et al., 2016; Fossataro et al., 2018), and it may correlate with other statements probing the experienced loss of the real hand (e.g., “It seemed like I couldn't really tell where my hand was”; Longo et al., 2008).

**TABLE 1:** Questionnaire items.

Item	Statement	Experience
S1	It seemed like the touch I felt was caused by the brush touching the rubber hand	Referral of touch
S2	It seemed like the rubber hand was my hand	Sense of ownership
S3	It seemed like I could have moved the rubber hand if I had wanted	Sense of agency
S4	It seemed like my real hand had disappeared	Disownership
S5	It seemed like the rubber hand was changing color	Control

Proprioceptive drift towards the rubber hand was assessed by placing a custom card “ruler” over the real and rubber hands, centered on the screen, from which participants reported the number that corresponded to their felt middle finger position. Eighteen different rulers were used—one for each measurement of proprioceptive drift during the experiment. Each was split into 29 rectangles of 1 cm width, with a number from 1 to 29 in each rectangle. The order of the numbers was randomized and different for each ruler, such that participants could not anchor on a single value across trials. The central rectangle was situated over the screen, such that 14 rectangles extended in the direction of the real hand, and 14 extended in the direction of the rubber hand. The rubber hand was obscured during the recording of proprioceptive drift and questionnaire responses.

#### 2.3.2 TMS and electromyography (EMG)

A custom script written in MATLAB 2018b (MathWorks, Inc.), using PsychToolBox (http://psychtoolbox.org/) and the HandLabToolBox (https://github.com/TheHandLab/HandLabToolBox), was used for signaling stroking of the real and rubber hand, as well as synchronizing TMS pulses. This script was also used for recording proprioceptive drift and questionnaire responses. The MAGIC toolbox (Saatlou et al., 2018) was used for triggering TMS.

For baseline and experimental data collection, TMS was applied at 110% of the resting motor threshold (RMT) using a Magstim BiStim^2^ and a 40 mm outer diameter figure‐of‐eight precision coil (The Magstim Company Ltd.) over the left primary motor cortex. Participants wore a lycra swimming cap to provide a uniform surface for stimulation. The coil was held manually by an experimenter during stimulation, and the Brainsight stereotactic neuronavigation system (Rogue Research Inc.) was used to ensure that the coil position and orientation remained consistent across conditions. Participants wore earplugs to protect against the noise of the TMS.

Surface EMG was recorded from the right FDI using DE‐2.1 Single Differential electrodes and the Delsys Bagnoli desktop system (Delsys Inc.). The recording area was cleaned with an alcohol wipe and the electrode was placed over the belly of the muscle. A reference electrode was placed on the left clavicle. The electrodes were secured with medical tape if necessary. The EMG signal was bandpass‐filtered between 20 and 450 Hz, amplified (gain = 1000), and sampled at 5000 Hz in Spike2 software (version 7.04) via a CED Micro1401‐3 data acquisition unit (Cambridge Electronic Design Limited).

### 2.4 Procedure

Participants were screened to ensure it was safe for them to undergo TMS (Rossi et al., 2009, 2011). As mentioned above, only participants susceptible to the RHI took part in the experiment as in della Gatta et al. (2016). To screen for susceptibility, participants were presented with two periods of 60 seconds of continuous stroking of the real and false right hand, synchronously and asynchronously, in a counterbalanced order (with the same spatial and temporal constraints as described for the main experiment below). During this time, they looked at the rubber hand. After each period of stroking, they were presented with three statements, in a random order, to which they were asked to provide their level of agreement on a scale of +3 (*strongly agree)* to −3 *(strongly disagree*). These statements addressed ownership over the rubber hand (“It seemed like the rubber hand was my hand”), referral of touch (“It seemed like the touch I felt was caused by the brush touching the rubber hand”), and a control statement (“It seemed like the rubber hand was changing color”). Between synchronous and asynchronous stroking periods, the participant viewed and moved their real hand to destroy any carry‐over effects. Participants were then asked to openly describe their experience in the two conditions.

Participants were accepted for the experiment if they provided a response greater than zero for the ownership statement in the synchronous condition and if their response was greater in the synchronous than in the asynchronous condition. They were excluded from testing if they failed to meet these criteria, if they provided a questionnaire response greater than zero for every statement in both conditions, if they openly reported that the synchronous and asynchronous stroking resulted in the same qualitative experience, or if they displayed confabulation for the control statement (i.e., vividly explaining how they observed the rubber hand changing color). In addition to this preregistered screening protocol, we also excluded four participants after starting the full experiment. One was excluded due to a hairstyle that made it impossible to place the TMS coil closely to the scalp, one was excluded after reporting not experiencing the RHI during experimental data collection, and two were excluded due to participant movement of the infrared markers used for neuronavigation.

Once suitability for TMS and illusion‐susceptibility was confirmed, we recorded the participant's RMT. The vertex was located by using a measuring tape to find the location halfway between both the two pre‐auricular points and the inion and nasion. From this location, we placed the coil on the left hemisphere 5 cm lateral and 1 cm anterior, from which we then localized the position over which we could detect MEPs in the FDI EMG trace. The handle of the coil was pointed in a posterior direction 45° from the midline. We increased stimulation intensity until MEPs with a peak‐to‐peak amplitude of > 0.05 mV were reliably observed and then reduced stimulation intensity until less than 10 out of 20 pulses induced an MEP with an amplitude > 0.05 mV. RMT was defined as this percentage of maximum stimulator output (MSO) plus 1 (Rossini et al., 2015). The mean ± SD RMT was 42.9% ± 5.85% MSO.

Once the RMT was found, we collected data for the amplitude of MEPs at baseline. Participants sat with their hands relaxed on the table. Both their hands and their upper body were covered with a cloth, and participants were asked to attend to a white fixation cross located on the center of the table (Figure 1). Fifteen pulses were applied with a random interval of 10–15 seconds between them.

We then collected MEPs for three conditions: one experimental and two control (Figure 1). In all conditions, participants’ real hands were hidden, with a cloth covering their body and upper arms. The real hand for which the RHI was induced was hidden behind the screen, whereas the opposite hand was on the table hidden by the cloth. However, only one rubber hand (left or right) was present on the table in any condition, to which the participant was asked to attend. In the experimental condition, where MEP amplitude was expected to be reduced according to the hypothesis of della Gatta et al. (2016), stroking was applied synchronously to the participant's right hand and a right rubber hand (rightSync; see below for further details). In the control conditions, stroking was applied synchronously to the participant's left hand and a left rubber hand (leftSync) or asynchronously to the participant's right hand and a right rubber hand (rightAsync). Both of these control conditions were reported by della Gatta et al. (2016), although in our case we tested the leftSync condition in a within‐participants fashion (without a corresponding asynchronous version, to avoid an exceptionally long experiment duration). There is no reason to expect corticospinal excitability to be reduced in the two control conditions, either because the illusion is not induced (rightAsync) or because the illusion is induced on the ipsilateral hand (leftSync). Using these two control conditions allowed us to ensure that any changes in MEP amplitude in the rightSync condition are both illusion‐ and hemisphere‐specific.

The three conditions were tested in three runs, repeated in a set order (e.g., ABC, ACB, BAC, etc.). The order was counterbalanced across participants. Within each run, participants first performed the proprioceptive drift task. The rubber hand was obscured, the ruler placed above the table, and participants were asked to verbally report the number under which they felt the position of their middle finger (pre‐test). The number of 1 cm squares from the position of the real hand to the position of the rubber hand was recorded. The rubber hand was then unobscured and the TMS component of the run began (Figure 1). This consisted of 10 trials, with a single pulse applied at the end of each trial. Within a single trial participants observed the rubber hand being stroked whilst their own hand was stroked for 12 seconds. Twelve seconds was chosen as this is in keeping with the paradigm presented by della Gatta et al. (2016), and earlier studies have found that the illusion is typically elicited within 10 seconds of repeated stroking of the type used in the present paradigm (Ehrsson et al., 2004; Lloyd, 2007). Participants were reminded to focus on the rubber hand when touches were applied.

Using a small brush, strokes were applied to the middle finger of the rubber hand, from the metacarpophalangeal to the distal interphalangeal joint, at a frequency of 0.5 Hz by an experimenter. That is, during the 12 seconds, six strokes were applied, each lasting 1 second (note that the preregistration erroneously stated this value as 1 Hz, when 0.5 Hz was the intended value). The experimenter timed the stroking based on an audio tone played in headphones via the TMS‐triggering computer. In synchronous conditions, stroking was also applied to the participant's own middle finger, matched as closely as possible to that performed on the rubber hand. In our preregistration, we planned that in the rightAsync condition, stroking of the rubber hand would be applied in a lateral to medial direction over the top of the hand (just below the metacarpophalangeal joints), during the “off” period of the real hand strokes. However, upon starting the experiment, we observed that this could interfere with the electrode placed on the FDI in some participants. As such, we decided in the rightAsync condition to apply touches to the two middle fingers purely out of phase, with touches applied to the real hand first (i.e., our asynchronous task only manipulated the relative timing of the seen and felt touches, which is also in line with the asynchronous control condition in many previous RHI studies). Following each 12 second stroking period, there was a 2 second pause, following which a pulse was applied. After a further 3 seconds, the next trial began. These small pauses allowed MEPs to be recorded without potential influence from the tactile stimulation. We know that the RHI is maintained for brief periods of at least 5 seconds after stroking ends, so there was no risk of the illusion being “lost” during these short periods without stroking (Abdulkarim et al., 2021). Pilot experiments confirmed that it was still possible to experience the illusion despite the brief muscular twitches in the hand caused by TMS in some individuals.

After 10 trials were performed, the rubber hand was obscured again, and the proprioceptive drift task (post‐test) was performed once again. Lastly, participants were verbally presented with the questionnaire statements in random order and responded with their level of agreement. There was a 5‐min break between each run to ensure corticospinal excitability returned to baseline, and test pulses were applied so that this could be confirmed by assessing MEP amplitude (as many as necessary to confirm, spaced at least 5 seconds apart). The participant also observed and moved their own hand during this 5‐min period, to ensure that the illusion was destroyed. This break also provided the opportunity to minimize participant fatigue. We planned to exclude participants if they were not able to keep their hands still while stroking was performed, but this was unnecessary. The entire experimental procedure, including screening, lasted approximately 2.5 to 3 h.

For one participant, a technical error meant that no behavioral data were collected for the first run of the leftSync condition, so this run was not used to calculate averages of behavioral responses for the condition, and nor was the EMG data for this run used.

### 2.5 Data analysis

A semi‐automated script written in Python 3 was used for data preprocessing. This script extracted questionnaire statements and proprioceptive drift for each block for each condition. Proprioceptive drift was defined as the post‐test minus pre‐test with positive values indicating a drift towards the rubber hand (which is the expected direction of drift in the case of an RHI; Abdulkarim & Ehrsson, 2016; Botvinick & Cohen, 1998; Tsakiris & Haggard, 2005). The median questionnaire response and mean proprioceptive drift for each condition for each participant were saved for statistical analysis. EMG data were filtered using a notch filter to remove 50 Hz line interference. MEPs were then extracted for the baseline and experimental conditions. MEP amplitude was defined as the difference between the maximum and minimum values of the EMG signal in the period 20 to 40 ms following the TMS pulse (i.e., peak‐to‐peak amplitude). MEPs with an amplitude < 0.05 mV were discarded since this would suggest an MEP was not induced (e.g., if the participant moved their head away from the coil prior to the pulse). Trials in which the difference between the greatest and smallest value exceeded 0.05 mV in the 100 ms prior to the TMS pulse were also excluded since this would suggest movement prior to the pulse occurring. To further exclude the influence of any possible head or hand movements time‐locked to the MEP pulse, or potential artifacts in the data, trials were also excluded if the amplitude of the MEP was greater than 2 SD away from the within‐condition mean. Finally, all trials were visually inspected for artifacts and excluded in those cases. Experimental and control condition MEPs were converted to a percentage of the mean MEP amplitude at baseline, and the mean per condition (30 MEPs across all runs) was saved for statistical analysis. Following preprocessing, we maintained 92.6% of baseline MEPs and 86.4% of experimental and control condition MEPs in total. Within conditions, we maintained 87.6% of MEPs in rightSync and rightAsync (mean of 26 trials per condition per participant) and 83.9% of MEPs in leftSync (mean of 25 trials).

In our preregistration, we stated that participants would be excluded entirely if less than 50% of their MEPs in any condition, or in the baseline, were excluded. However, one participant met this criterion for only the leftSync condition, which was not analyzed for our key hypothesis test. We decided to maintain this participant for any analysis that did not involve the leftSync condition. We removed one participant who had too few trials in the rightAsync condition following data processing. We also planned to exclude participants if they provided a response greater than zero for every questionnaire statement in every condition since this could suggest unusually strong suggestibility or otherwise unreliable questionnaire responses. However, this was not necessary.

Statistical tests were performed in JASP (JASP Team, 2021). Participant responses to questionnaire statements were compared across conditions using Wilcoxon signed‐rank tests. Comparisons for proprioceptive drift and MEP amplitude were tested for normality using a Shapiro–Wilk test. In the case of deviations from normality in any of these comparisons, we compared all conditions using Wilcoxon signed‐rank tests. Otherwise, we used paired samples *t*‐tests. Planned comparisons were made only between rightSync and rightAsync, and rightSync and leftSync (i.e., to test our experimental condition against the two controls). Based on a large number of previous studies from many different laboratories, we predicted that proprioceptive drift and responses to statements S1 and S2 would be greater in rightSync, compared to rightAsync (e.g., Abdulkarim & Ehrsson, 2016; Lloyd, 2007; Tsakiris & Haggard, 2005), which would indicate successful induction of the RHI. On the basis of a few previous studies (e.g., Fossataro et al., 2018; Lane et al., 2017; Longo et al., 2008; Reader et al., 2021), we also hypothesized that the responses to S3 and S4 would be greater in rightSync, compared to rightAsync. In the rightSync condition, we expected positive affirmative responses to statements S1 and S2, while responses to S3 and S4 may be negative for most participants (though still greater than in the rightAsync condition). We expected proprioceptive drift and responses to questionnaire statements S1–S4 would be broadly similar between the rightSync and leftSync conditions, although we cannot exclude the possibility that the induction of the illusion on the non‐dominant left hand might result in a greater proprioceptive drift and a stronger sense of ownership over the fake (Dempsey‐Jones & Kritikos, 2019; Niebauer et al., 2002; but see Ocklenburg et al., 2011; Smit et al., 2017). We did not expect any differences between conditions in control statement S5, and any such differences may be interpreted as cognitive bias or an effect of suggestibility. Statistical tests were one‐tailed where strong predictions in one direction can be made on the basis of the previous literature (S1, S2, S3, S4, proprioceptive drift when comparing rightSync and rightAsync); otherwise, they were two‐tailed.

In our preregistration we proposed that, if we replicate the results of della Gatta et al. (2016), we would expect that MEPs have a smaller amplitude relative to baseline in the rightSync condition compared to the two control conditions. However, the primary analysis for assessing whether we replicated the effect of della Gatta et al. (2016) was the comparison between rightSync and rightAsync, since they observed a statistically significant difference in MEP amplitude between synchronous and asynchronous stroking of the rubber hand. In the absence of a statistically significant reduction in MEP amplitude relative to baseline in rightSync, compared to rightAsync, we planned to assess the level of evidence in favor of the null hypothesis (no difference between rightSync and rightAsync) using a one‐sided Bayesian paired samples *t*‐test (Rouder et al., 2009; alternative hypothesis = rightSync < rightAsync). We planned to compare the two conditions using a normally distributed prior centered on the effect size 0.74 (reported by della Gatta et al., 2016), with an SD of half this effect size (Dienes, 2014). We planned to collect further data until we reached 30 participants in total or the Bayes factor provided consistent reasonable evidence in favor of the null hypothesis over the alternative hypothesis (BF_10_ < 0.333; Jarosz & Wiley, 2014). Evidence in favor of the null hypothesis was considered consistent if the Bayes factor remained below the threshold for three consecutive participants.

Had we observed a statistically significant difference in MEP amplitude between rightSync and rightAsync, we planned to assess correlations between the magnitude of illusion effects (difference between rightSync and rightAsync in proprioceptive drift and statements S1–S4) and the difference between MEP amplitude across conditions. This was not necessary (see Results), but we provide the following preregistered analysis plan for transparency. We planned to perform this analysis with two‐sided Bayesian Kendall rank correlations (van Doorn et al., 2018), using a default stretched beta prior width of 1, zero‐centered (given that we had no strong predictions regarding the size of any possible effect). We also planned to report the robustness of the Bayes factor: the maximum possible Bayes factor and the associated stretched beta prior width. We planned to collect further data until we reached 30 participants in total, or the Bayes factor provided consistent reasonable evidence in favor of the null hypothesis over the alternative hypothesis (BF_10_ < 0.333), or the alternative hypothesis over the null hypothesis (BF_10_ > 3) for three out of the five correlations. Evidence in favor of either hypothesis was to be considered consistent if the Bayes factor remained above the threshold for three consecutive participants.

## 3 RESULTS

### 3.1 Behavioral results

The level of agreement with questionnaire statements was significantly greater in rightSync, compared to rightAsync for items S1 to S4 that reflect the RHI, with at least 83% of participants providing an increased response in the rightSync condition: S1 (W = 171, *p* < .001 [one‐tailed], *r* = 1, 95% CI = [1, ∞]), S2 (W = 153, *p* < .001 [one‐tailed], *r* = 1, 95% CI = [1, ∞]), S3 (W = 134, *p* < .001 [one‐tailed], *r* = .971, 95% CI = [0.927, ∞]), S4 (W = 125.5, *p* = .00152 [one‐tailed], *r* = .846, 95% CI = [0.648, ∞]).

There was also a significant difference between rightSync and rightAsync for control item S5 (W = 21, *p* = .0340, *r* = 1, 95% CI = [1, 1]). Despite this, only 33% of participants provided an increased response for rightSync and most ratings were negative; thus, the difference between the conditions simply reflected differences in how certain some participants were in rejecting this control statement. There was no significant difference in agreement to questionnaire statements between rightSync and leftSync: S1 (W = 10.5, *p* = 1, *r* = 0, 95% CI = [−0.712, 0.712]), S2 (W = 31.5, *p* = .714, *r* = .145, 95% CI = [−0.503, 0.689]), S3 (W = 26.5, *p* = .668, *r* = .178, 95% CI = [−0.505, 0.724]), S4 (W = 17, *p* = .944, r = −.0556, 95% CI = [−0.682, 0.618]), S5 (W = 9.5, *p* = .915, *r* = −.0952, 95% CI = [−0.756, 0.611]; Table 2).

**TABLE 2:** Questionnaire responses.

Item	Experience	Summary responses (condition, percentile)								
		rightSync			rightAsync			leftSync		
		25th	50th	75th	25th	50th	75th	25th	50th	75th
S1	Referral of touch	2	3	3	−3	−2	−1	2	3	3
S2	Ownership	1.25	2	3	−2.75	−1.5	−0.25	2	2	2.5
S3	Agency	1	1	2.75	−3	−1.5	1	1	2	2
S4	Disownership	1.25	2	2	−2.75	−1.5	0.75	1	2	2
S5	Control	−3	−2.5	−1.25	−3	−3	−2.25	−3	−2	−1

Proprioceptive drift was significantly greater in rightSync (mean ± SE = 1.50 ± 0.431 cm), compared to rightAsync (0.704 ± 0.373 cm), with 83% of participants showing an effect in this direction, W = 143.5, *p* = .00607 (one‐tailed), *r* = .678, 95% CI = [0.366, ∞]. There was no significant difference between rightSync and leftSync (1.62 ± 0.332 cm), W = 66.0, *p* = .636, r = −.137, 95% CI = [−0.591, 0.383].

In summary, both the questionnaire results and the proprioceptive drift results indicated that the RHI was elicited as expected in the two synchronous conditions (rightSync and leftSync) and abolished in the asynchronous condition.

### 3.2 TMS results

**FIGURE 2:**
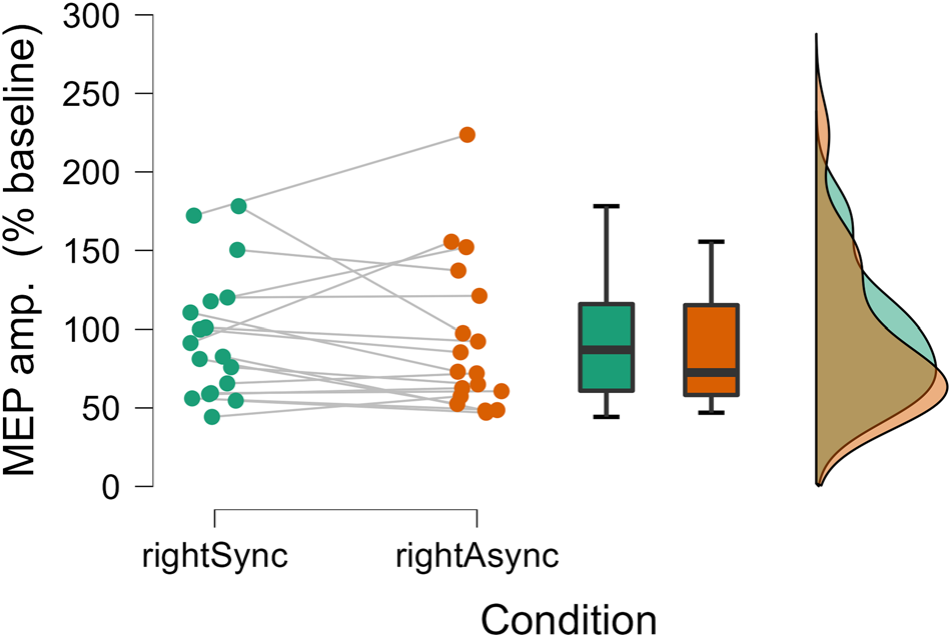
Individual datapoints, box‐and‐whisker plots, and distributions for motor‐evoked potential (MEP) amplitude (% of baseline) in rightSync and rightAsync.

There was no significant difference in MEP amplitude as a percentage of baseline between rightSync (95.5 ± 9.44%) and rightAsync (91.8 ± 11.4%), *t*(17) = 0.483, *p* = .636, *d* = 0.114, 95% CI = [−0.351, 0.576]; Figure 2). Only 44% of participants showed a reduced MEP amplitude in rightSync, compared to rightAsync. Similarly, there was no significant difference between rightSync and leftSync (105 ± 9.49%), *t*(16) = −1.02, *p* = .324, *d* = −0.247, 95% CI = [−0.726, 0.240], with 56% of participants showing a reduced MEP amplitude in rightSync (Supporting Information Figure S1). Mean MEPs for the baseline and each condition are displayed in Supporting Information Figure S2.

Since we observed no statistically significant difference between rightSync and rightAsync after collecting data for 18 participants, we performed our planned one‐sided Bayesian *t*‐test to evaluate the evidence in favor of the null hypothesis. We observed that BF_10_ = 0.0696, indicating that the data were over 14 times more likely under the null hypothesis than the alternative hypothesis (1/BF_10_). This result was consistent over three consecutive participants. To ensure that this result was not due to an overestimate of the possible effect size, we decided post hoc to repeat the analysis with the prior distribution situated on a smaller effect size. We set the mean of the distribution to *d* = 0.37 (i.e., half of the original effect size estimate), with an SD of half of this size. We observed that BF_10_ = 0.221, once again indicating greater support for the null hypothesis.

## 4 DISCUSSION

Several studies have proposed that the RHI can alter the excitability or connectivity of the motor system (Dilena et al., 2019; Golaszewski et al., 2021). We performed a conceptual replication of a key study by della Gatta et al. (2016) with the aim of verifying the influence of the RHI on corticospinal excitability as reflected in MEP amplitude. We also hoped to better understand the factors that contribute to this potential physiological change during the illusion. However, contrary to the findings of della Gatta et al. (2016), we did not observe a reduction in MEP amplitude for the hand over which the illusion was induced. This result can be interpreted in three ways. Firstly, reductions in MEP amplitude may be small or not reliable. Secondly, the reduction in MEP amplitude reported by della Gatta and colleagues may have arisen due to methodological choices rather than due to an effect of the RHI. Thirdly, there may be no true effect of the RHI on MEP amplitude, and the previously reported result may be a false positive.

Regarding the first interpretation, it remains feasible that the RHI does alter MEP amplitude, yet the true effect is very small. The effect size reported by della Gatta et al. (2016) when comparing MEP amplitude between synchronous and asynchronous conditions was relatively high (*dz* = 0.85), which may be an overestimation of the population effect. It is plausible then that our study was not adequately powered to detect smaller population effects. However, a Bayesian analysis using a prior distribution situated on an effect size of *d* = 0.37 still suggested that the data were more likely under the null hypothesis than the alternative. It is possible that the true effect could be smaller still, but this would bring into question the importance of such a physiological change (discussed in more detail below). Furthermore, it is noteworthy that less than half of our participants showed a reduced MEP amplitude in the rightSync condition, compared to rightAsync. This occurred despite the behavioral results showing clear and significant differences in the RHI measures between the key synchronous and asynchronous conditions at the group level, with all participants affirming that they experienced the illusion (although subjective report from a single subject on a questionnaire cannot be taken as conclusive evidence that the person actually perceived the illusion, since questionnaire ratings may not be well protected against compliance, cognitive bias, suggestibility, or differences in decision criteria; Chancel & Ehrsson, 2020; Chancel, Ehrsson, & Ma, 2022; Lush, 2020; Lush et al., 2020; Reader, 2022; Slater & Ehrsson, 2022). This indicates that a reduction in MEP amplitude may not be a reliable outcome of the RHI. Despite these two possibilities, it is worth stating that a single replication study may not provide an effective verification of the presence of an effect, particularly if neither study is adequately powered to detect the true effect (Hedges & Schauer, 2019).

It is also possible that the effect reported by della Gatta et al. (2016) arose from methodological choices rather than manipulation of body ownership (or any other phenomena specifically arising from the RHI). For example, della Gatta et al. (2016) applied their synchronous and asynchronous conditions in single runs with over double the duration that we did (∼340 vs. ∼170 s). In addition to the key differences in MEP amplitude between the synchronous condition and the asynchronous condition and baseline, they also found that the reduction in MEP amplitude during the synchronous condition was more pronounced over time (although it is not clear from their article whether this is an interaction effect with no comparable results in the asynchronous condition). One possibility is that such extended illusion induction is a requirement for changes in MEP amplitude, and it is the reduction in MEP amplitude at later timepoints that drives the differences between the synchronous condition and asynchronous condition/baseline. Why such changes in excitability would only emerge after an extended illusion experience is not clear. Some explanation may be provided by results indicating that MEP amplitude is increased when visual attention is directed away from one's hand, compared to towards it (Bell et al., 2018). As such, it is possible that the changes in corticospinal excitability reported by della Gatta et al. (2016) are due to differences in attention across conditions that were facilitated by their longer runs, where attention may be more likely to wane over time if the task is not engaging. That is, more consistently maintained visual attention towards the limb one feels is one's own during synchronous stimulation could reduce MEP amplitude compared to the less engaging asynchronous condition (where the observed hand is not perceived as one's own) and baseline (where observation of the hand is not possible).

Conversely, in our experiment, the duration of visuotactile stimulation was adequate to elicit strong agreement with RHI statements, though perhaps with the benefit of similar attentional demands across conditions given our shorter runs and a balanced design. Furthermore, even if differences in attentional demands are not an adequate explanation, and our RHI induction procedure was simply not long enough to alter MEP amplitude, this would mean it is unlikely that reductions in MEP amplitude arise due to the subjective RHI or disownership of the real hand, the latter proposed by della Gatta and colleagues. Such experiences were reported quite strongly in our sample, despite the relatively shorter runs. Moreover, if changes in MEP amplitude develop long after the illusion has been elicited and maintained for two minutes, it cannot be related to the causal mechanisms of the illusion but may instead reflect a consequence of the illusion on the motor cortex that develops slowly as a result of prolonged illusion exposure (see below). However, it is worth pointing out that neither our study nor that of della Gatta et al. (2016) controlled for visuospatial attention, making it difficult to truly evaluate the degree to which this may explain our different results. Future studies could better control for attention, for example by having participants perform a demanding attentional task (e.g., Gentile et al., 2013) during the TMS procedure. Similarly, monitoring gaze and fixation could be important.

A further interpretation of our data is that the previous finding by della Gatta et al. (2016), describing a reduction in MEP amplitude, is a false positive. This interpretation could potentially bring into question broader claims about the influence of body ownership manipulations on motor cortical activity, but evidence in favor of the proposal is mixed. Karabanov et al. (2017) did not observe any change in MEP amplitude following the induction of a moving version of the RHI, but the sample for this part of their experiment consisted of only seven participants. Functional magnetic resonance imaging (fMRI) studies do not report changes in motor cortical activity during the RHI, but then the blood‐oxygen‐level‐dependent signal reflects overall population synaptic activity in an area (including the input; Logothetis et al., 2001), which is different to the measure of excitability facilitated by TMS. Conversely, a recent study using TMS combined with electroencephalography (EEG) reported a reduction in TMS‐induced evoked potentials from electrodes over the sensorimotor cortex region that seems to support a reduction of motor cortical excitability during illusory limb ownership using virtual reality (Casula et al., 2022). However, since EEG has limited spatial resolution, it remains unclear if the modulation of the EEG responses observed was driven primarily from changes in motor cortical excitability of the upper‐limb representation of the primary motor cortex as reported by della Gatta et al. (2016). TMS‐induced changes in EEG activity may also have a different physiological basis to the MEPs recorded in our study and that of della Gatta et al. (2016), with the latter reflecting the excitability of the corticospinal tract captured in the descending effect of TMS on spinal motor neurons. Regardless, “illusory amputation” induced by virtual reality has also been reported to result in a reduction of MEP amplitude (Kilteni et al., 2016; though we note that this paradigm is quite different from the RHI, and effects were not observed for the FDI). Furthermore, MEP amplitude is only one measure of motor cortical excitability. Reductions in short‐interval intracortical inhibition, an alternative measure, have also been reported to occur during the RHI (Alaydin & Cengiz, 2021). Additionally, there are studies indicating alterations in parietal‐motor connectivity (Isayama et al., 2019; Karabanov et al., 2017), which further support the occurrence of physiological changes in the motor system following body ownership manipulation. Thus, even if changes in MEP amplitude are not reliably observed, other physiological aspects of the motor system may still be affected.

On the balance of evidence then, the first interpretation above, that true effects of the RHI on MEP amplitude are small or are not reliable, is perhaps the most feasible. The exact cause of such small effects, which may occur only in some participants, remains to be verified, especially given the aforementioned limitations of the “disownership” hypothesis proposed by della Gatta et al. (2016). It remains unclear how changes in MEP amplitude might relate to the changes in motor cortical excitability (e.g., short‐interval intracortical inhibition) or connectivity reported in other studies. It is also unclear whether such effects can tell us much about the potential role of body ownership in motor control more generally. One possibility is that changes in MEP amplitude, if they can occur but were not detected in our study, are simply a side effect of increased inhibitory output to the motor cortex from posterior parietal regions involved in multisensory body perception (Casula et al., 2022). Such an inhibitory influence could arise in some individuals purely from the strong structural and functional connectivity between the motor cortex and posterior parietal regions, the latter playing an important role in both motor control (Rizzolatti & Luppino, 2001) and multisensory integration during the RHI (Chancel, Iriye, & Ehrsson, 2022; Ehrsson et al., 2004). In any case, one must be cautious in interpreting changes in MEP amplitude in functional terms (Bestmann & Krakauer, 2015). Indeed, we have previously observed that body ownership illusions do not convincingly influence reaction time or maximal speed and acceleration of brisk finger movements, which speaks against behaviorally relevant changes in motor circuit excitability (Reader & Ehrsson, 2019; Reader et al., 2021). Ultimately, and as others have noted, body ownership illusions appear to have a potentially complex effect on motor cortex excitability (Alaydin & Cengiz, 2021). It therefore seems likely that more research on motor cortical (or corticospinal) excitability is necessary to uncover exactly how and why alterations in body ownership influence the state of the motor system. Examining other measures of corticospinal excitability could be informative. For example, some prior work has successfully examined the cortical silent period during the RHI (Schütz‐Bosbach et al., 2009), although effects were associated with an interaction between action attribution and body ownership rather than body ownership per se. In addition, combining TMS and movement may help to verify whether any changes in motor excitability are functional.

Importantly, it should also be noted that neither our results nor those of della Gatta et al. (2016) provide evidence for the proposal that the primary motor cortex *contributes* to changes in body ownership perception through a reduction in motor cortical activity (Casula et al., 2022; Fossataro et al., 2018) since this would presumably require a reduction in excitability *prior* to the illusion occurring. This was not measured in our experiment or that performed by della Gatta et al. (2016). Regardless, the motor system may play an important role in body ownership, primarily through the involvement of non‐primary motor areas. For example, activity in the premotor cortex and cerebellum is reported in fMRI studies of the RHI (e.g., Brozzoli et al., 2012; Ehrsson et al., 2005, 2004). Although these activations have been interpreted as reflecting multisensory integration in the previous literature (because the participants do not move, the neural responses follow the spatial and temporal principles of multisensory integration, and work in non‐human primates has described multisensory neuronal populations in these areas; Ehrsson et al., 2004; Fang et al., 2019; Gentile et al., 2013; Graziano, 1999; Graziano et al., 2000), these regions are also critical for motor control (Manto et al., 2012; Rizzolatti & Luppino, 2001). Rather than reflecting a role of the primary motor cortex, changes in body ownership seen after limb immobilization (Burin et al., 2017) or in hemiplegic patients (Burin et al., 2015) could stem from neural plasticity or tissue damage to these multisensory areas or their anatomical connections with other nodes in the cortical and subcortical circuits that control movement (but see Fossataro et al., 2018).

Finally, we address some potential limitations in our work that may restrain our interpretation of the results. Notably, our experiment took much longer to complete than that of della Gatta et al. (2016). Despite providing breaks, we cannot exclude that changes in alertness across the session may have increased the variability of MEP amplitude (Noreika et al., 2020; but see Cleland et al., 2023; Cuypers et al., 2014). Furthermore, we used relatively low‐intensity stimulation (110% RMT), which may be more susceptible to the intra‐subject variability normally observed in MEP amplitudes (e.g., Darling et al., 2006; Kiers et al., 1993). Indeed, the within‐participant coefficient of variation (standard deviation divided by mean) for MEPs in our sample was similar to that typically observed at 110% of RMT (approx. 0.6, see Supporting Information; Darling et al., 2006). A high level of MEP variability may have made it more challenging to find between‐condition differences due to increased measurement noise. Although we did use the same stimulus intensity as della Gatta et al. (2016), a “true” effect will be harder to detect in noisy data. However, if the proposed effect is much smaller than the naturally occurring variability in MEP amplitude induced using a typical stimulation intensity, then this may actually further support our claim that changes in corticospinal excitability during the RHI are smaller than previously estimated.

In summary, we failed to observe a reduction in MEP amplitude during the RHI. We propose that the most plausible explanation for this is that such changes are unlikely to be large or reliable. If they do occur, they may be a minor side effect of altered activity in multisensory parietal regions and should be interpreted with caution. Further examination of the influence of body ownership alterations on different measures of corticospinal excitability is likely to be informative, however, especially if they can better control for attentional demands. More work is also necessary to verify the functional relevance of altered motor cortical excitability and connectivity during body ownership illusions.

## AUTHOR CONTRIBUTIONS

Arran T. Reader, Victoria S. Trifonova, and H. Henrik Ehrsson designed the experiment. Arran T. Reader, Sara Coppi, and Victoria S. Trifonova piloted the experiment and collected data. Arran T. Reader and Sara Coppi analyzed the data. All authors contributed to writing the manuscript.

## CONFLICT OF INTEREST STATEMENT

The authors declare no conflicts of interest.

### PEER REVIEW

The peer review history for this article is available at https://publons.com/publon/10.1002/brb3.3211.

## Supporting information


**FIGURE S1** Individual datapoints, box‐and‐whisker plots, and distributions for MEP amplitude (% of baseline) in rightSync and leftSync.
**FIGURE S2** Mean MEPs for baseline and experimental conditions. Note that these mean time series have been baseline‐corrected to begin with an amplitude of 0 mV.

## Data Availability

The data that support the findings of this study are openly available in the OSF at https://doi.org/10.17605/OSF.IO/T8x4B.
