# Supplementary material for: No reduction in motor‐evoked potential amplitude during the rubber hand illusion
Source: Brain Behav. 2023 Aug 7;13(10):e3211. doi: 10.1002/brb3.3211 (PMC10570491; doi:10.1002/brb3.3211)
Supplement: Supplementary file 1 — FIGURE S1 Individual datapoints, box‐and‐whisker plots, and distributions for MEP amplitude (% of baseline) in rightSync and leftSync. FIGURE S2 Mean MEPs for baseline and experimental conditions. Note that these mean time series have been baseline‐corrected to begin with an amplitude of 0 mV. [file BRB3-13-e3211-s001.pdf]

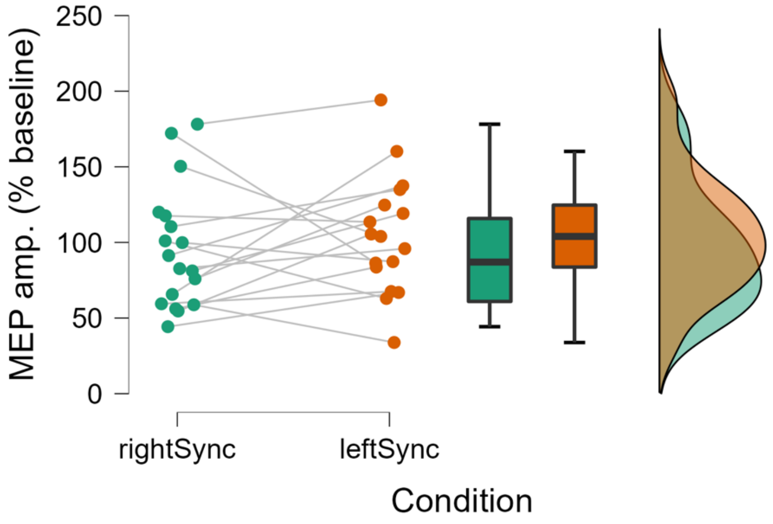

Figure S1: Individual datapoints, box-and-whisker plots, and distributions for MEP amplitude (% of baseline) in rightSync and leftSync

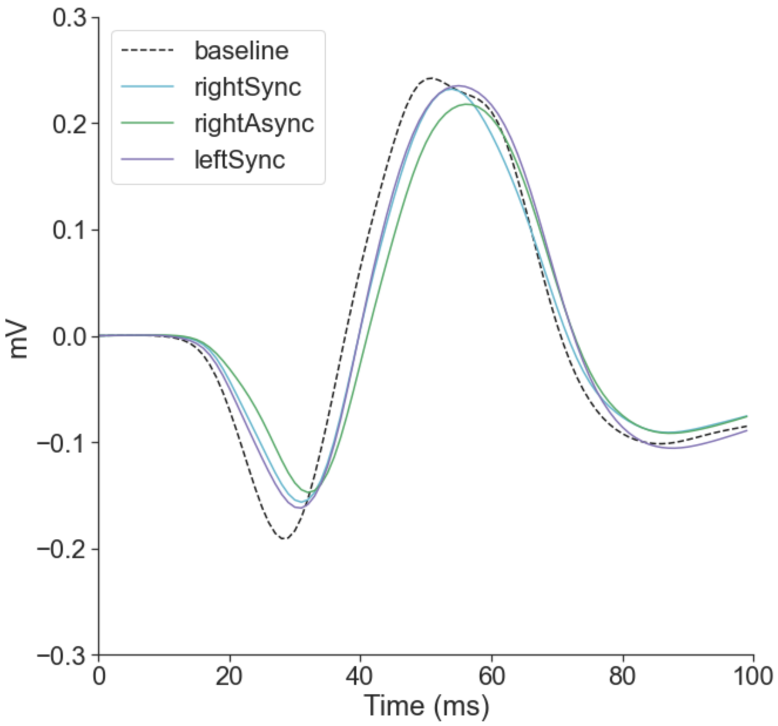

Figure S2: Mean MEPs for baseline and experimental conditions. Note that these mean timeseries have been baseline-corrected to begin with an amplitude of 0 mV.

Supplemental post hoc analysis

To examine the coefficient of variation (CV) for MEP amplitudes, the standard deviation was divided by the mean for each condition for each participant. This provided a single CV value per participant for each condition. The grand mean±SE CV was 0.624±0.0418.

Comparing CV between conditions using a Bayesian paired samples t-tests with an uninformed Cauchy prior (scale 0.707, zero-centred) revealed that the data were at least 2.6 times more likely under the null hypothesis (no difference) than the alternative (condition 1 ≠ condition 2):

RightSync (0.656±0.0541) compared to rightAsync (0.610±0.0574), BF_{10} = 0.327

RightSync compared to leftSync (0.589±0.149), BF_{10} = 0.384

RightAsync compared to leftSync, BF_{10} = 0.267
